# Effect of different carbon dioxide (CO_2_) flows on trapping *Aedes albopictus* with BG traps in the field in Zhejiang Province, China

**DOI:** 10.1371/journal.pone.0243061

**Published:** 2020-12-01

**Authors:** Yuyan Wu, Jinna Wang, Tianqi Li, Qinmei Liu, Zhenyu Gong, Juan Hou

**Affiliations:** Zhejiang Provincial Center for Disease Control and Prevention, Hangzhou City, Zhejiang Province, China; Fundacao Oswaldo Cruz Instituto Rene Rachou, BRAZIL

## Abstract

Carbon dioxide (CO_2_) attracts host-seeking adult mosquitoes; this fact is exploited for mosquito monitoring, which is important for evaluating the effects of mosquito-control operations. A field experiment was designed to explore the relationship between the CO_2_ flow rate and the trapping effect of BG traps. The aim was to select an appropriate flow rate for monitoring *Aedes albopictus*. Six sampling sites were selected for field experiments in Hangzhou city, Zhejiang Province, China. A total of six CO_2_ flow rates (0.00 L/min, 0.075 L/min, 0.15 L/min, 0.30 L/min, 0.60 L/min and 1.20 L/min) were tested to compare their effects on mosquito trapping. The catches were performed in six trapping periods between 15:30 and 18:30, and each catch period lasted 0.5 h. A total of 3068 adult mosquitoes were captured at six sampling sites in six days using BG traps (with BG-Sweetscent), among which 86.96% were *Ae*. *albopictus*. The total number of *Ae*. *albopictus* (males and females) captured at a flow rate of 0.00 L/min was significantly lower than the numbers captured at 0.075 L/min, 0.15 L/min, 0.30 L/min, 0.60 L/min and 1.20 L/min (*P*<0.001, *P*<0.001, *P*<0.001, *P*<0.001, and *P*<0.001 respectively). The total number of *Ae*. *albopictus* captured and the number of *Ae*. *albopictus* females captured increased with increasing CO_2_ flow and peaked at 0.3 L/min, above which these capture numbers did not increase significantly. In conclusion, the appropriate CO_2_ flow rate for monitoring *Ae*. *albopictus* with BG traps was 0.3 L/min.

## Introduction

Mosquito-borne diseases, such as dengue fever, Zika disease, chikungunya fever and West Nile fever, have caused substantial damage to human health and economic development in recent years [1–5]. In mainland China, 22,188 dengue fever cases were reported via the National Notifiable Diseases Reported System (NNDRS) in 2019. *Aedes albopictus* is the primary vector of dengue virus, Zika virus and chikungunya virus in mainland China and is widely distributed in China [6–8]. To date, only larval surveillance of *Ae*. *albopictus* has been conducted in most areas of China, and the larval density of *Ae*. *albopictus* does not necessarily predict its adult density [9, 10]. The density of adult *Ae*. *albopictus*, especially adult *Ae*. *albopictus* females, is directly associated with the spread of mosquito-borne diseases. Therefore, monitoring adult *Ae*. *albopictus* is of vital importance to effectively evaluate vector density and the effects of vector management [9, 11].

Currently, few methods are available for the surveillance of *Ae*. *albopictus* adults in China. The human landing catch method (HLC) is considered to be the “gold standard” because of its high effectiveness in attracting host-seeking mosquitoes [12]. Though this method is sensitive and efficient, it exposes field workers the potential risk of mosquito-borne pathogens, including dengue and Zika viruses [11, 12]. Moreover, human-bait attraction bias is also an inevitable disadvantage to the human-bait catch method [13]. If the human baits are different, the density of mosquitoes might not be comparable among different times or places. Recently, the human-baited double net (HDN) trap was recommended as a supplement for *Ae*. *albopictus* monitoring by the Chinese Center for Disease Control and Prevention. The HDN trap protects the human bait from hungry mosquitoes with an inner net. The collector, wearing long-sleeved clothing, collects mosquitoes between the two nets. Though these traps are safe, the efficiency of the human bait is weak because the two box nets restrict individual variation by limiting excess attractant emanations from the host inside the inner net. Furthermore, it is very difficult for mosquitoes to enter the gap between the two nets [11]. Thus, HDN traps are not effective for collecting *Ae*. *albopictus*, and their monitoring efficiency is low. A useful monitoring method combining safety, efficiency and stability is crucial for the surveillance of *Ae*. *albopictus* adults in China.

The BG trap is a tool used for routine mosquito surveillance of *Ae*. *albopictus* and *Ae*. *aegypti* in North America, Singapore and Australia; it has high efficiency in catching *Ae*. *albopictus* according to some previous studies [14–16]. It also showed strong potential for emergency monitoring when mosquito-borne diseases occurred [17]. BG traps use carbon dioxide (CO_2_) and attractants that mimic human scents to attract mosquitoes. However, a previous study focused on other traps reported that different CO_2_ flows might lure different species of mosquitoes and that the efficiency of trapping mosquitoes varied [18–21]. Little is known about the trapping efficiency of different CO_2_ flows for trapping mosquitoes, especially *Ae*. *albopictus*, in BG traps. Few studies focused on what level of CO_2_ flow is suitable for trapping *Ae*. *albopictus*, especially *Ae*. *albopictus* females (the real culprits in spreading diseases, whose density is of great concern), in BG traps have been conducted in China. Thus, in this study, we assessed the efficiency of different CO_2_ flows for trapping adult mosquitoes in BG traps in Zhejiang Province. Our objective was to explore the relationship between CO_2_ flow and trapping efficiency and then to select an appropriate flow for monitoring *Ae*. *albopictus* adults.

## Methods and materials

### Ethics statement

No permits were required for the described field studies. These studies did not involve endangered or protected species, and the mosquito collections performed at the sampling sites were consented to by the site owners at each location.

### Study area

This study was conducted in summer 2018 in Hangzhou city, Zhejiang Province, China. Hangzhou city is located in the north of Zhejiang Province, which has a subtropical monsoon climate. Summers are hot and rainy, with temperatures ranging from 19–35°C and relative humidity ranging from 50%-94%. A total of 6 field monitoring sites (from A to F) distributed throughout two districts representing urban (from A to D) and suburban (E and F) environments were selected for mosquito monitoring. The distance between any two sampling sites was at least 200 m. Details of the site characteristics are shown in Table 1.

**Table 1:** Geographical information for the six mosquito sampling sites.

Site ID	District	Type of environment	Coordinates
A	JG	Residential neighborhood	30°17′51.50″N,120°12′4.45″E
B	JG	Institution	30°17′50.23″N,120°12′1.39″E
C	JG	Park	30°17′49.15″N,120°11′59.07″E
D	JG	Green area	30°17′47.24″N,120°12′4.97″E
E	BJ	Green area	30°9′59.32″N,120°9′25.47″E
F	BJ	Residential neighborhood	30°10′2.32″N,120°9′31.20″E

Abbreviations: JG, Jianggan District; BJ, Binjiang District.

### Mosquito sampling

BG trap catches were conducted at six field sites on random windless sunny days in June 2018. A special experimental design was used in this study to effectively compare the trapping effects of different CO_2_ flows by controlling the impact of other factors, including the sampling site and trapping period. Six CO_2_ flow groups (0.00 L/min, 0.075 L/min, 0.15 L/min, 0.30 L/min, 0.60 L/min and 1.20 L/min) were established, and the 0.00 L/min CO_2_ flow group was regarded as the control group. All six CO_2_ flow groups were tested in turn, trapping mosquitoes at one site per day. The catches per 0.5-h trapping period were performed for each CO_2_ flow group between 15:30 and 18:30 in the afternoon; a total of six catches were conducted a day at one sampling site. The CO_2_ flow sequences were changed among sites on each sampling day and among days at each sampling site (Table 2).

**Table 2:** The CO_2_ flow setup at the six sampling sites and six time periods.

Day 1	Site			Time			
		15:30–16:00	16:00–16:30	16:30–17:00	17:00–17:30	17:30–18:00	18:00–18:30
	A	0.00	0.075	0.15	0.30	0.60	1.20
	B	0.075	0.15	0.30	0.60	1.20	0.00
	C	0.15	0.30	0.60	1.20	0.00	0.075
	D	0.30	0.60	1.20	0.00	0.075	0.15
	E	0.60	1.20	0.00	0.075	0.15	0.30
	F	1.20	0.00	0.075	0.15	0.30	0.60
Day 2	Sites	15:30–16:00	16:00–16:30	16:30–17:00	17:00–17:30	17:30–18:00	18:00–18:30
	A	0.075	0.15	0.30	0.60	1.20	0.00
	B	0.15	0.30	0.60	1.20	0.00	0.075
	C	0.30	0.60	1.20	0.00	0.075	0.15
	D	0.60	1.20	0.00	0.075	0.15	0.30
	E	1.20	0.00	0.075	0.15	0.30	0.60
	F	0.00	0.075	0.15	0.30	0.60	1.20
Day 3	Sites	15:30–16:00	16:00–16:30	16:30–17:00	17:00–17:30	17:30–18:00	18:00–18:30
	A	0.15	0.30	0.60	1.20	0.00	0.075
	B	0.30	0.60	1.20	0.00	0.075	0.15
	C	0.60	1.20	0.00	0.075	0.15	0.30
	D	1.20	0.00	0.075	0.15	0.30	0.60
	E	0.00	0.075	0.15	0.30	0.60	1.20
	F	0.075	0.15	0.30	0.60	1.20	0.00
Day 4	Sites	15:30–16:00	16:00–16:30	16:30–17:00	17:00–17:30	17:30–18:00	18:00–18:30
	A	0.30	0.60	1.20	0.00	0.075	0.15
	B	0.60	1.20	0.00	0.075	0.15	0.30
	C	1.20	0.00	0.075	0.15	0.30	0.60
	D	0.00	0.075	0.15	0.30	0.60	1.20
	E	0.075	0.15	0.30	0.60	1.20	0.00
	F	0.15	0.30	0.60	1.20	0.00	0.075
Day 5	Sites	15:30–16:00	16:00–16:30	16:30–17:00	17:00–17:30	17:30–18:00	18:00–18:30
	A	0.60	1.20	0.00	0.075	0.15	0.30
	B	1.20	0.00	0.075	0.15	0.30	0.60
	C	0.00	0.075	0.15	0.30	0.60	1.20
	D	0.075	0.15	0.30	0.60	1.20	0.00
	E	0.15	0.30	0.60	1.20	0.00	0.075
	F	0.30	0.60	1.20	0.00	0.075	0.15
Day 6	Sites	15:30–16:00	16:00–16:30	16:30–17:00	17:00–17:30	17:30–18:00	18:00–18:30
	A	1.20	0.00	0.075	0.15	0.30	0.60
	B	0.00	0.075	0.15	0.30	0.60	1.20
	C	0.075	0.15	0.30	0.60	1.20	0.00
	D	0.15	0.30	0.60	1.20	0.00	0.075
	E	0.30	0.60	1.20	0.00	0.075	0.15
	F	0.60	1.20	0.00	0.075	0.15	0.30

We prepared six BG traps of the same type with different CO_2_ flows (0.00 L/min, 0.075 L/min, 0.15 L/min, 0.30 L/min, 0.60 L/min and 1.20 L/min) in six positions with similar mosquito densities at each sampling site on each day. The first trap was turned on at 15:30. After half an hour, the second BG trap was turned on and the first trap was turned off. This procedure was repeated for the next four BG traps. The trapping sequences for the BG traps with different CO_2_ flows are summarized in Table 2. All BG traps were positioned 50 m apart. The study ended when all the CO_2_ flows were tested in turn in six time periods and at six field sites. The study lasted for six days.

### *BG trap* catches

**Fig 1:**
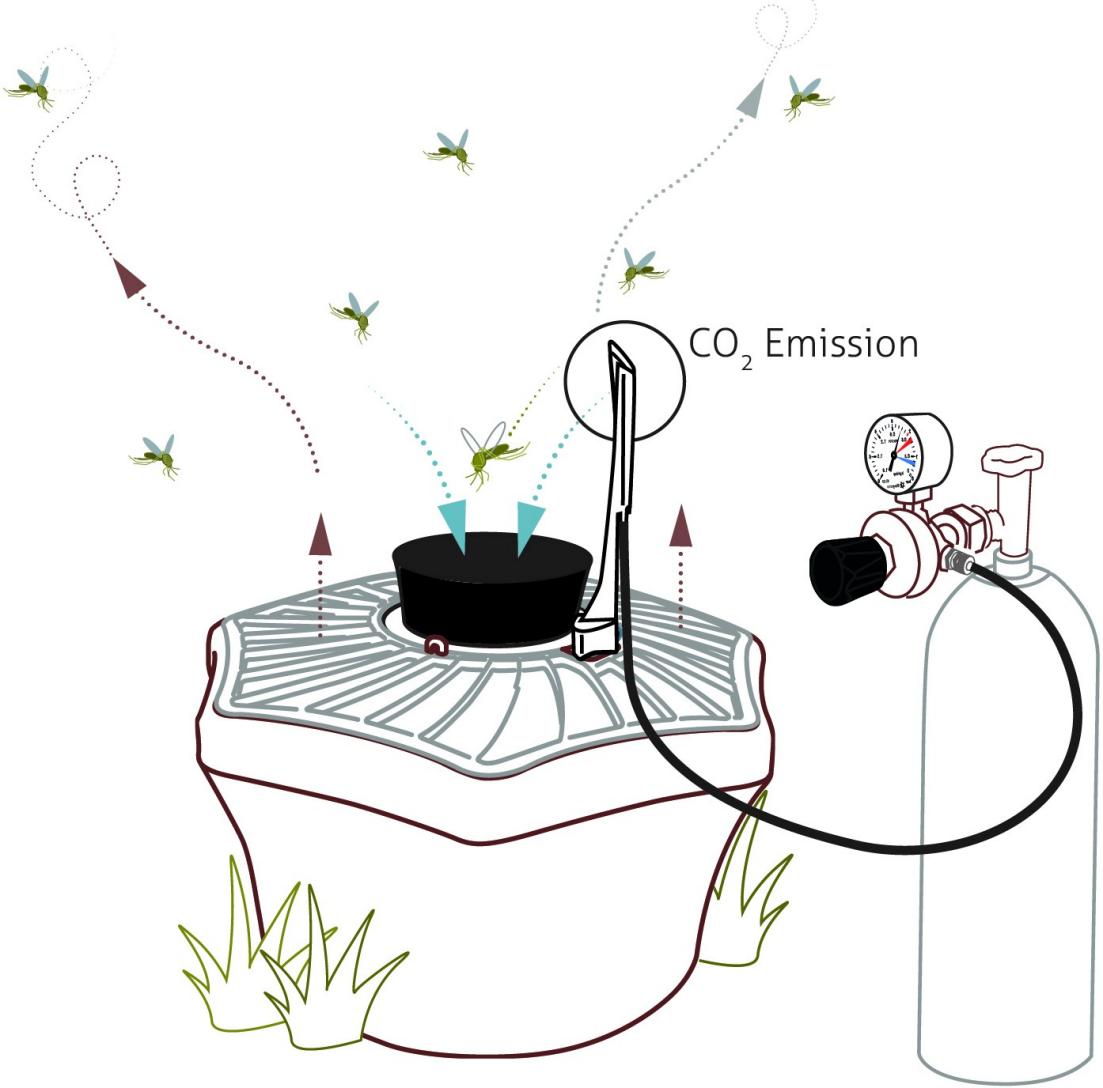
The overall schematic diagram of the BG-Mosquitaire CO_2_ trap.

BG traps (version: BG-Mosquitaire CO2) developed by BioGents GmbH Company (Regensburg, Germany, SN: 00040145) were used in this research. Each black funnel trap was placed on the ground with the trap mouth opening upwards in lightly shaded places sheltered from the wind. BioGents GmbH Company's self-developed mosquito attractant (BG-Sweetscent) was placed in the funnel trap, the power supply was connected, and then the carbon dioxide valve was opened (Fig 1). A standardized mass flowmeter (version 4100 series, TSI, America) was used to ensure the precision of the CO_2_ flow during the experiment.

The mosquitoes collected were taken to the Zhejiang Provincial Center For Disease Control And Prevention laboratory, killed by freezing, and identified using taxonomic keys [22]. Approximately 36 groups of mosquitoes were collected on each day, and a total of 216 groups of mosquitoes were obtained.

### Statistical analysis

Data preparation and statistical analyses were performed using SPSS (version 23.0) [23]. Generalized linear mixed models (GLMMs) were used to analyze the trapping effect of different CO_2_ flows on the numbers of total *Ae*. *albopictus* (males and females) and *Ae*. *albopictus* females caught per trapping period based on negative binomial regression. The dependent variables were modeled via GLMMs controlling for independent random variables (“days”, in this case) to test the statistical significance of fixed independent variables (“CO_2_ flows”, “sites” and “hours”, in this case). The means and standard errors associated with the GLMMs were calculated.

## Results

### General observations

**Fig 2:**
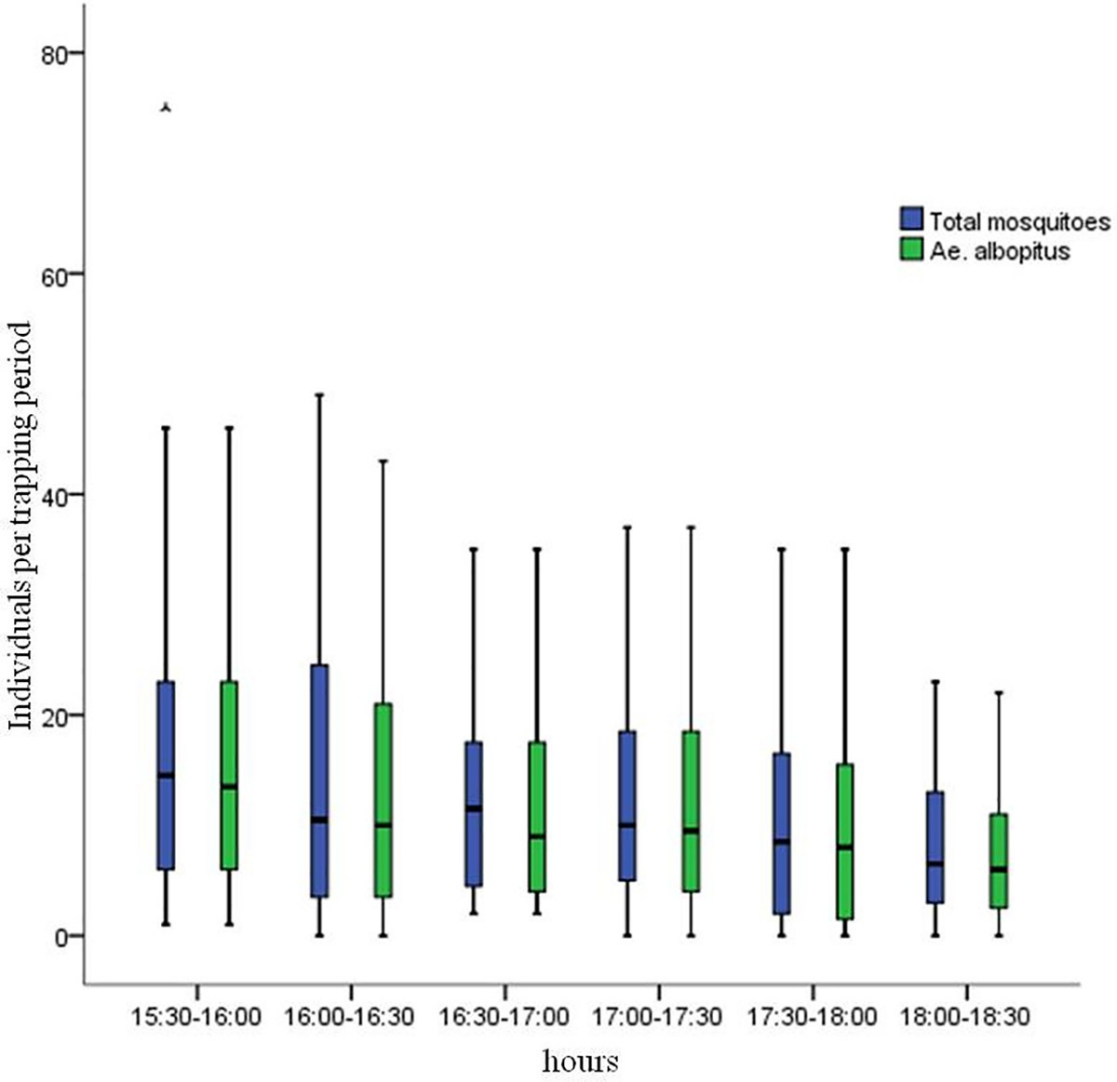
Differences in the number of individuals trapped (total mosquitoes and *Ae*. *albopictus*) among time periods.

A total of 3068 adult mosquitoes were captured at the six sampling sites on six days with BG traps. Three mosquito species were identified, and the individuals collected were predominantly *Ae*. *albopictus* (2668, 86.96%). The other two mosquito species were of the *Culex pipiens* complex (mainly *C*. *quinquefasciatus* and *pallens*, 319, 10.40%) and *C*. *tritaeniorhynchus* (81, 2.64%). The median number of mosquitoes yielded by the BG traps at different flow rates for six sites ranged from 8.00 to 32.00, and the difference was statistically significant (GLMM, *F*_(5,200)_ = 15.310, *P*<0.001). Among the six sampling sites, the most mosquitoes were caught at sites C and F, and the fewest were caught at sites A and B (Table 3). The mosquitoes trapped at different time points are presented in Fig 2. Statistically significant variations in the total mosquito catches were found among the different hours of the day (GLMM, *F*_(5,200)_ = 8.514, *P*<0.001). The number of mosquitoes observed per trapping period at 15:30–17:30 was significantly higher than that at 18:00–18:30, and more mosquitoes tended to be caught at 15:30–16:00 (Table 4). As the main mosquito species captured, *Ae*. *albopictus* (males and females) showed the same trends as the total mosquitoes, which peaked at 15:30–16:00 (Table 4, Fig 2).

**Table 3:** Statistical differences between the number of trapped individuals per trapping period among the six trapping sites.

Site	Estimate	SE	t	p	95% CI	
					Lower	Upper
A	-1.051	0.177	-5.955	<0.001*	-1.399	-0.703
B	-0.869	0.174	-0.500	<0.001*	-1.212	-0.527
C	0.081	0.166	0.488	0.626	-0.246	0.407
D	-0.008	0.166	-0.049	0.961	-0.336	0.319
E	-0.177	0.167	-1.059	0.291	-0.507	0.153
F^#^	0^#^	/	/	/	/	/

Mean +/−SE differences in the least squares means associated with the mixed linear models for the number of individuals per trapping period among the six trapping sites. Estimate: differences in the least squares means, SE: standard error, DF: degrees of freedom, t: t-value, p: p value.

# Site F was selected as the baseline.

*significant differences were found.

**Table 4:** Statistical differences between the number of individuals trapped per trapping period among the six trapping periods.

Time	Total mosquitoes				*Ae*. *albopictus*			
	estimate	SE	t	p	estimate	SE	t	p
15:30–16:00 vs 16:00–16:30	5.376	2.761	2.078	0.039	0.434	0.172	2.529	0.012
15:30–16:00 vs 16:30–17:00	7.809	2.754	2.835	0.005	0.603	0.713	3.481	0.001
15:30–16:00 vs 17:00–17:30	8.028	2.757	2.912	0.004	0.615	0.173	3.550	0.000
15:30–16:00 vs 17:30–18:00	10.663	2.829	3.770	0.000	0.914	0.176	5.187	0.000
15:30–16:00 vs 18:00–18:30	11.593	2.872	4.036	0.000	1.082	0.178	6.076	0.000
16:00–16:30 vs 17:30–18:00	4.927	1.948	2.529	0.012	0.480	0.179	2.684	0.008
16:00–16:30 vs 18:00–18:30	5.857	1.953	2.999	0.003	0.648	0.181	3.586	0.000
16:30–17:00 vs 18:00–18:30	3.784	1.658	2.283	0.023	0.480	0.182	2.635	0.009
17:00–17:30 vs 18:00–18:30	3.565	1.628	2.189	0.030	0.467	0.182	2.567	0.011

Mean +/−SE differences in the least squares means associated with the generalized mixed linear models for the number of total individuals and *Ae*. *albopictus* per trapping period among the six trapping periods. Only significant differences are shown. Estimate: differences in the least squares means, SE: standard error, DF: degrees of freedom, t: t-value, p: p value.

### The relationship between CO_2_ flows and mosquito catches/species

**Fig 3:**
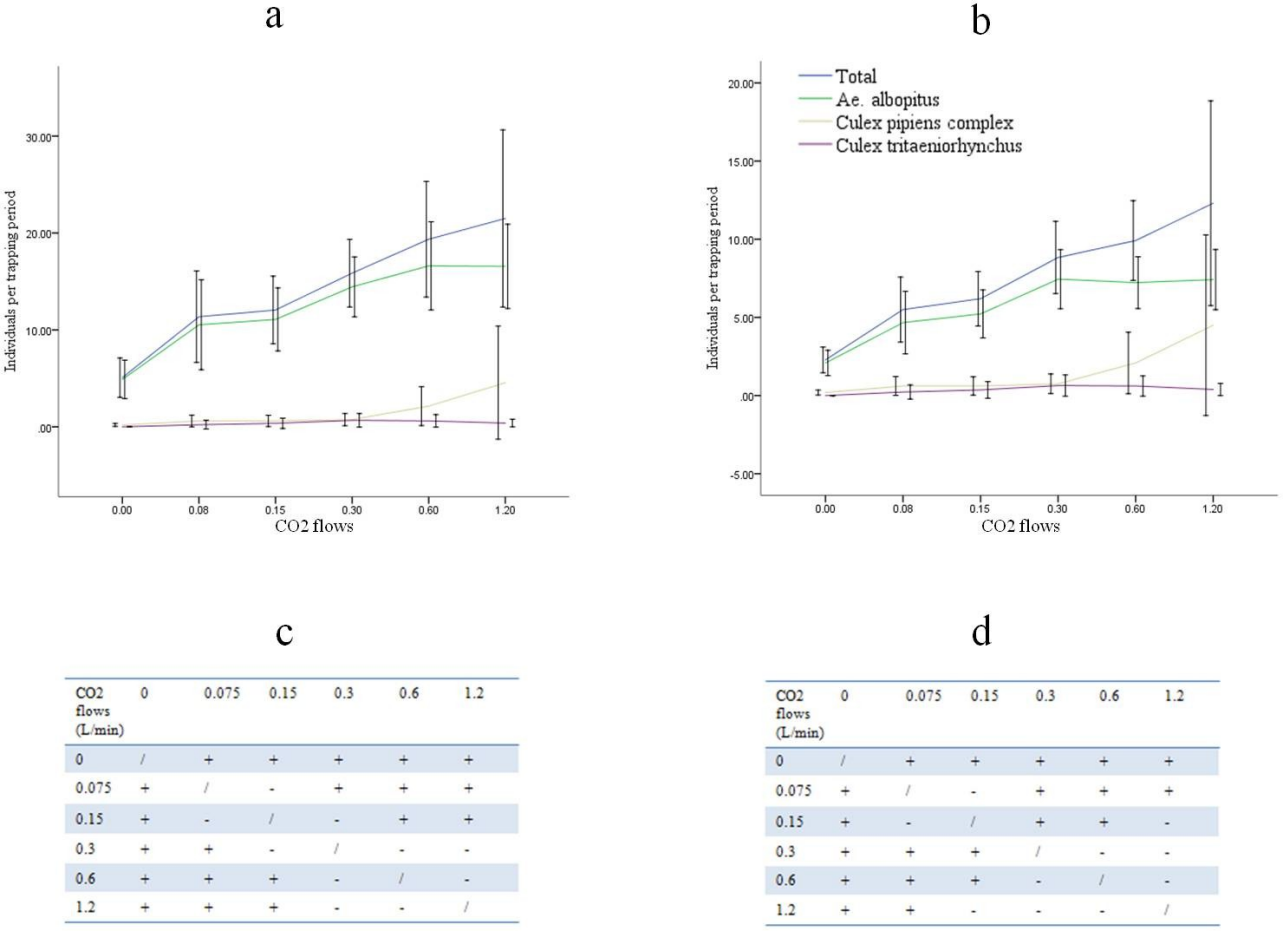
**The mosquitoes captured at different CO**_**2**_ **flows and the significant differences of *Ae*. *albopictus* trapped.** Values are the medians±95% confidence intervals. Differences in the least squares means were associated with the mixed linear models for the number of *Ae*. *albopictus* and *Ae*. *albopictus* females captured per trapping period among the six CO_2_ flows. **a** The total number of mosquitoes captured. **b** The number of female mosquitoes captured. **c** The significant differences between the numbers of *Ae*. *albopictus* trapped among the six CO_2_ flows. **d** The significant differences between the numbers of *Ae*. *albopictus* females trapped among the six CO_2_ flows.+ Significant differences were found,−There was no significant difference.

**Fig 4:**
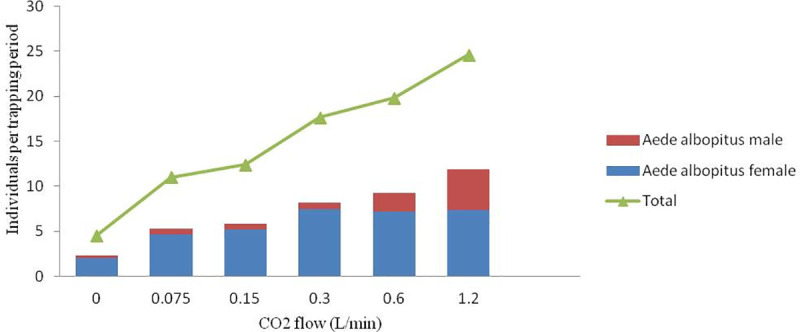
Number of trapped individuals among the six CO_2_ flows.

Approximately 176 total *Ae*. *albopictus* were collected in six days in the control group alone, which was significantly fewer than the number collected in the test groups (0.075 L/min, 0.15 L/min, 0.30 L/min, 0.60 L/min and 1.20 L/min)(GLMM, t = -4.038 P<0.001, t = -5.193 P<0.001, t = -7.046 P<0.001, t = -7.388 P<0.001, and t = -7.137 P<0.001, respectively). With the increase in CO_2_ flow, the number of collected *Ae*. *albopictus* increased. When the CO_2_ flow was 1.20 L/min, the capture rate of *Ae*. *albopictus* was 14.83 per hour, which was 3.39 times that at 0.00 L/min (4.17 per hour). The capture rate of *Ae*. *albopictus* significantly increased after 0.15 L/min and reached a peak at 0.6 L/min, after which the number of *Ae*. *albopictus* captured per trapping period remained stable (Fig 3A). However, no significant differences were found between the number of *Ae*. *albopictus* trapped per trapping period among the 0.3 L/min, 0.6 L/min and 1.2 L/min flow rates (Fig 3C). The number of *Ae*. *albopictus* females trapped showed a similar trend as that the total number of *Ae*. *Albopictus* trapped, which peaked at 0.3 L/min (Fig 3B and 3D). The number of *Ae*. *albopictus* males caught increased consistently with increasing CO_2_ flow (GLMM, *F*_(5,200)_ = 10.060, *P*<0.001) and the male ratio (defined as the number of *Ae*. *albopictus* males caught divided by the total number of *Ae*. *albopictus* caught per trapping period) increased obviously at flow rates higher than 0.6 L/min (Fig 4).

The capture rate of *Culex pipiens quinquefasciatus* obviously increased after the CO_2_ flow was 0.3L/min and continued increased thereafter (Fig 3A). The *Culex pipiens quinquefasciatus* captured at 0.00L/min was statistically fewer than that of 0.60L/min and 1.20L/min (GLMM, t = -2.118, P = 0.035; t = -3.046, P = 0.003, respectively). But it was not statistically fewer than groups with CO_2_ flow 0.075L/min, 0.15L/min and 0.3L/min (GLMM, t = -1.309, P = 0.192; t = -1.245, P = 0.215; t = -1.775, P = 0.077, respectively). Due to relatively small sample size, the catch of *Culex tritaeniorhynchus* continued to be at a low level (Fig 3A).

## Discussion

In this study, six CO_2_ flows (0.00 L/min, 0.075 L/min, 0.15 L/min, 0.30 L/min, 0.60 L/min and 1.20 L/min) applied with BG traps were compared for their effects on capturing mosquitoes, especially *Ae*. *albopictus*, in Zhejiang Province. The results indicated that the CO_2_ flow was significantly associated with the trapping efficiency of BG traps for *Ae*. *albopictus*. However, thresholds were observed for both the total *Ae*. *albopictus* (0.6 L/min) and the *Ae*. *albopictus* females (0.3 L/min) captured, over which the numbers of total *Ae*. *albopictus* and *Ae*. *albopictus* females captured per trapping period remained stable. Though the total number of *Ae*. *albopictus* captured per trapping period peaked at 0.6 L/min, no significant difference was found among the numbers of *Ae*. *albopictus* captured at 0.6 L/min, 1.2 L/min and 0.3 L/min. On the basis of the numbers of mosquitoes captured at different CO_2_ flows, we suggest that a suitable CO_2_ flow for monitoring *Ae*. *albopictus* with a BG trap is 0.3 L/min. A similar study on this issue was conducted by Ge et al. [9] in Beijing city, China; they reported that 6 L/min was the best CO_2_ flow rate for trapping *Ae*. *albopictus*. The discrepancy between these recommended CO_2_ flows for trapping *Ae*. *albopictus* might be attributed to the different types of traps used (MT-1 trap vs BG trap).

Previous studies have indicated that the BG trap is an effective, safe and stable alternative catch method for monitoring *Ae*. *albopictus* [14–16]. However, it remains unclear which CO_2_ flow rate is most suitable for monitoring *Ae*. *albopictus* with BG traps. In this study, the number of *Ae*. *albopictus* captured per trapping period at 0.00 L/min was significantly lower than those at 0.075 L/min, 0.15 L/min, 0.3 L/min, 0.6 L/min and 1.2 L/min. The number of captured mosquitoes was significantly associated with the CO_2_ flow rate, which is consistent with some previous studies [24]. As monitoring was carried out at the peak activity time for *Ae*. *albopictus* in Zhejiang Province, 86.96% of the captured mosquitoes were *Ae*. *albopictus*. Unlike the number of captured *Ae*. *albopictus* females, the number of captured *Ae*. *albopictus* males continued to increase with the increase in CO_2_ flow rather than constantly maintaining a low density level. This phenomenon might be explained by mosquito behavior. To mate with females, male mosquitoes tend to locate potential hosts and remain nearby to increase their chances of encountering females [25–29]. The more CO_2_ is released, the more likely it seems that hosts are present, which attracts more males [30]. The trapping efficiency for *Culex pipiens* in the control group was significantly lower than that at 0.6 L/min and 1.2 L/min, but not significantly different from that at 0.075 L/min, 0.15 L/min or 0.30 L/min. Due to the relatively small sample size, the catch of *Culex tritaeniorhynchus* was consistently low.

This study was conducted using BG traps, and the results should not be extrapolated to other traps. The trapping results showed an interesting trend for the *Culex pipiens* complex, indicating that further studies with more CO_2_ flow groups, more time periods (*Culex pipiens* complex activity peaks after sunset in Zhejiang Province, at approximately 19:00) and more sample sizes could be conducted to explore trapping effects.

## Conclusion

The current study demonstrated that CO_2_ flow was significantly associated with the trapping efficiency of BG traps for *Ae*. *albopictus*. The appropriate CO_2_ flow for monitoring *Ae*. *albopictus* with a BG trap was 0.3 L/min.
